# Effects of Methionine on Milk Performance and Milk Constituents of Lactating Donkeys

**DOI:** 10.3390/ani14203027

**Published:** 2024-10-19

**Authors:** Fei Huang, Xinyi Du, Zongjie Ma, Guiqin Liu, Changfa Wang, Miaomiao Zhou

**Affiliations:** College of Agriculture and Biology, Liaocheng Research Institute of Donkey High-Efficiency Breeding and Ecological Feeding, Liaocheng University, Liaocheng 252000, China; huangfei080616@163.com (F.H.); duxinyi1289@163.com (X.D.); 18764659740@163.com (Z.M.); guiqinliu@lcu.edu.cn (G.L.); wangchangfa@lcu.edu.cn (C.W.)

**Keywords:** lactating donkeys, methionine, lactation performance, metabolomics

## Abstract

This study investigated the effects of adding methionine (Met) in milk production and the milk and blood metabolites of lactating donkeys. Eighteen healthy multiparous donkeys in early-stage lactation were selected for this study. The donkeys were randomly divided into three groups and fed diets with different levels of Met: control group (C, Met 0 g/d), Met group I (M1, Met 5 g/d), and Met group II (M2, Met 15 g/d). The total duration of the experiment was 5 weeks. Donkey milk and blood samples were collected at the end of the experiment. The milk yield and composition, milk, and serum metabolites were analyzed. The results showed that the addition of 5 g of Met significantly increased milk yield as well as the milk composition contents of protein, fat, lactoferrin, polyunsaturated fatty acid (PFA), solids, and solids-not-fat (SNF) (*p* < 0.05). Significant differences in metabolites were detected among the different samples of milk (*p* < 0.05). The addition of Met increased the levels of milk metabolites, such as myristic acid, d-glutamine, l-aspartic acid, and LPS 16:0. A total of 753 metabolites were detected in the serum, including 17 differential metabolites between C and M1 and 48 differential metabolites between C and M2. The levels of serum metabolites, such as l-ascorbate, inositol, and l-lysine, were up-regulated by the addition of Met (*p* < 0.05). The above results indicated that Met increased donkey milk production and milk composition yield and improved milk metabolites by regulating serum metabolites. These results provide a foundation for improving the nutritional needs of lactating donkeys and the nutritional regulation of donkey milk synthesis.

## Simple Summary

Donkey milk has a nutritional value similar to that of human milk. Methionine (Met) is an essential amino acid for lactating animals. The addition of dietary Met can improve lactation performance in dairy cows. However, the effects of Met on lactating donkeys have not been investigated. This study investigated the effects of the addition of different concentrations of Met on the milk performance and milk metabolites of lactating donkeys. The results showed that the addition of dietary Met improved the milk yield and milk component production of donkeys as well as altering the milk metabolites. This study provides a basis for the nutritional regulation of milk synthesis and a method for improving the lactation performance of donkeys.

## 1. Introduction

There are great differences in the nutritional compositions of milk produced by different animals [1,2]. Donkey milk is often considered to be the closest to human milk in terms of composition [3]. Research has shown that donkey milk contains several functional nutritional factors, including lysozyme, lactalbumin, lactoferrin, and immunoglobulins, which make it a high-quality milk source with nutrients similar to those found in human milk [4].

Donkey milk has been shown to enhance immunity, alter metabolisms, and beneficially alter gut microbiota [5]. In addition, at this stage, infant formulas are commonly made from cow’s milk, but developing a cow’s milk protein allergy has always been a problem that cannot be ignored (as it significantly increases the probability of dental caries in young children) [6]. Studies have shown that donkey milk, which has an 82.6–88% tolerance rate, is superior to cow milk in the production of infant formula and can be used for babies who have a cow milk protein allergy [7]. Infant intolerance to formula milk is a common issue that can cause gastroesophageal reflux (GER) in preterm infants [8]. Studies have shown that donkey milk, as opposed to bovine milk, can reduce the incidence of GER in preterm infants [9]. Donkey milk is suitable for infant nutrition due to its hypoallergenic nature; it contains high levels of niacin, which has a lipid-lowering effect, as well as high levels of vitamin B6 and folate, which contribute to child growth and development [10]. These properties make it a valuable option for infant nutrition.

A donkey’s nutrition, lactation duration, and breed are examples of elements that might impact the quality of milk. A previous study found that the type of roughage (maize stover, wheat husk, or straw) affected the lipid and volatile organic compound profile of donkey milk [11]. This finding demonstrated that providing sufficient dietary crude protein for lactating donkeys increased milk production [12]. Renu et al. (2023) found that in colostrum–early lactation, early–mid-lactation, and mid–late lactation, there were 24, 10, and 18 differently expressed metabolites, respectively [11]. Another study investigated how the stage of lactation affected the contents of the milk produced by grazing donkeys. As lactation progressed, the number of total solids, solids-not-fat (SNF), and nitrogenous fractions in the milk decreased [13]. Ante et al. analyzed the milk components of Istrian donkeys and Littoral Dinaric donkeys and reported notable differences in lactose and milk output, milk protein, and fat between the different breeds [14].

Lysine and Met are the two most important essential amino acids for lactating animals. In dairy cow research, Met is considered a limiting amino acid [15]. A previous study demonstrated how supplementing dairy cow diets with 2.34% rumen-protected Met increased the percentages of milk protein and fat [16]. Also, in nutritional trials with Holstein cows, it was found that, in comparison to control groups, feeding meals containing lysine and rumen-protected Met increased milk fat, lactose, real protein, and energy production [17]. Additionally, a study reported an increase in milk protein yield of 2.23 g after the addition of Met [18]. The addition of n-acetyl-l-Met also increased the milk yield of dairy cows [19].

Donkey milk is a high-quality milk source. However, there is little research on the regulation of donkey milk synthesis. Therefore, this study investigates the effects of Met supplementation on donkey milk composition and serum metabolites and analyzed the regulatory mechanism.

## 2. Materials and Methods

### 2.1. Animal Ethics

The animal experiments in this study were approved by the animal care and use committee of Liaocheng University (Liaocheng, Shandong, China) (2022111002).

### 2.2. Experimental Design

Eighteen healthy multiparous lactating Dezhou donkeys (the female donkeys had already given birth to 2–3 foals) with an average of 71.2 ± 2.6 d milk days and an average bodyweight of 273.4 ± 30 kg were used in this study. Each donkey was housed in a separate pen, with foal (average weight 71.6 ± 8.97 kg) and mother donkey pairs kept together. The total duration of the experiment was 5 weeks, with a pre-feeding period of 1 week. Three groups consisting of six pairings each were formed from the eighteen mother–child donkey pairs: the control group (C, Met 0 g/d), M1 group (Met, 5 g/d), and M2 group (Met, 15 g/d). The donkeys were raised in a partially closed shelter on a farm in Liaocheng City, Shandong Province, China. Water and the same diet of grass hay (6 kg) supplemented with 2.5 kg of concentrate per head per day were provided to all nursing donkeys. Foals were supplemented with 1 kg of concentrate/head per day, and the donkey foal’s concentrate feed was given in two separate troughs from the dam so that the donkey foal was not able to eat the feed in the dam’s trough during feeding. The concentrate composition and nutrient levels are shown in Table 1. The nutritional composition of concentrate feeds is based on dry matter.

### 2.3. Milk and Blood Collection and Foal Weight Data Collection

At the end of the experimental period, milk and blood were collected. Donkeys in lactation were manually milked twice a day at 11 a.m. and 3 p.m. during which time milk samples were collected (milk samples were taken at every instance of milking). The samples were frozen in liquid nitrogen and kept at −80 °C until examination. Donkey milk output was recorded. Foals were removed from their mother for 4 h before milking.

Blood was collected on the morning of the last day of the experiment (fasting for 12 h before collection). Samples were collected before the foals were weighed to avoid contamination from stress. Using an anticoagulant tube, blood samples were drawn from the jugular veins of the female donkeys. The blood was then centrifuged at 3500 rpm/min for 10 min to extract the upper serum. The serum samples were then frozen in liquid nitrogen and stored at −80 °C until analysis.

### 2.4. Measurement of Donkey Milk Composition

The lactose, fat, protein, SNF, solid, urea, lactoferrin, and polyunsaturated fatty acid (PFA) compositions of donkey milk were measured using CombiScope FTIR300 (Delta Instruments B.V., Drachten, The Netherlands).

### 2.5. Liquid Chromatography–Mass Spectrometry Metabolomics Analysis

The experimental method was referred to Wang et al. [20]. After thawing at room temperature, eighteen samples were carefully poured, 100 µL at a time, into 1.5 mL centrifuge tubes. After that, samples were mixed with 400 µL of a 1:1 *v*/*v* methanol/acetonitrile extraction solution. Three 10 min ultrasonic extractions were carried out on ice following vortexing. For thirty minutes, the samples were left to stand at −20 °C. Using a chilled centrifuge set at 4 °C, the supernatant was separated by centrifugation at 2675 rpm for 15 min. An LNG-T88 fast centrifugal concentration drier was then used to lyophilize the supernatant. Following lyophilization, a 100 µL reconstitution solution (a 1:1 *v*/*v* mixture of acetonitrile and water) was added, and the mixture was then transferred to a vial for LC-MS analysis. Using triple quadrupole time-of-flight (TOF) equipment in conjunction with ultra-high-pressure liquid chromatography, all samples were examined. Using a BEH C18 column, the chromatographic separation was carried out at 40 °C. The pretreatment plasma samples were injected with a volume of 20 µL. Both the 0.1% formic acid aqueous solution (solvent A) and acetonitrile/isopropanol (*v*/*v*, 1:1) with 0.1% formic acid (solvent B) comprised the two mobile phases. The gradient of the mobile phase (A:B) elution is given below, and the flow rate was 0.40 mL/min. Here, 80%: 20% for 0 to 3.0 min, 5%: 95% for 3.0 to 9.0 min, 5%: 95% for 9.0 to 13.0 min, 95%:5% for 13.0 to 13.1 min, and 95%: 5% for 13.1 to 16.0 min are the different ratios of 95%: 5% for certain time periods. On a triple TOF mass spectrometer equipped with an electrospray ionization source that could operate in both positive and negative ion mode, the mass spectral analysis and tandem mass spectral analysis were carried out. The energy of the impact, the injection voltage, and the capillary voltage were 6 eV, 40 V, and 1.0 kV, respectively. The temperatures of the ion source and desolvation were 120 °C and 500 °C, respectively. The mass data were taken between the mass-to-charge ratio (*m*/*z*) range of 50–1000, the nitrogen flow rate was 900 L/h, and the instrument resolution was 30,000. Throughout the analytical run, aliquots of all the plasma samples were mixed to create quality control (QC) samples, which were injected at regular intervals to assess the stability of the analytical system.

### 2.6. Bioinformatics and Statistical Analysis

Duncan’s multiple range tests and one-way ANOVA tests analyzed the data using SAS software (sas 9.2, sas Institute Inc., Cary, NC, USA). The values are presented as means ± standard deviations. Superscript-differentiated bars indicate significant differences (*p* < 0.05). To identify and screen differential metabolites, non-targeted metabolomics was employed with the parameters of VIP > 1.0, fold change (FC > 1.2), and FC < 0.833 with *p*-value < 0.05 [21]. Systematic pathway annotation was performed for all identified metabolites, and differential analysis and KEGG analysis were performed for all differential metabolites. Stability is important in the overall detection process; the Pearson correlation coefficient R2 between QC samples was between 0.992 and 0.996, which is very close to 1 [22].

## 3. Results

### 3.1. Effects of Met Supplementation on Milk Performance of Lactating Donkeys

The daily milk yield and the weight gain of the foals are shown in Table 2. The addition of Met increased milk production in lactating donkeys and increased weight gain in foals. Group M1 produced considerably more milk in comparison to the other groups (*p* < 0.05). The weight gain of donkey foals was greatest in the M2 group (*p* < 0.05).

The milk composition content and yield are shown in Table 3. The addition of Met had significant effects on milk composition content and yield. Compared to group C, the milk contents of lactoferrin, PFA, and solids were significantly greater (*p* < 0.05) in group M1. Furthermore, the milk concentrations of protein, lactoferrin, PFA, solid, and SNF were considerably greater in group M2 than in group C (*p* < 0.05). Overall, group M1 had significantly greater yields of milk composition, milk protein, fat, lactose, PFA, lactoferrin, solids, and SNF than the other groups (*p* < 0.05).

### 3.2. Effects of Met Supplementation on Donkey Milk Metabolites

**Figure 1:**
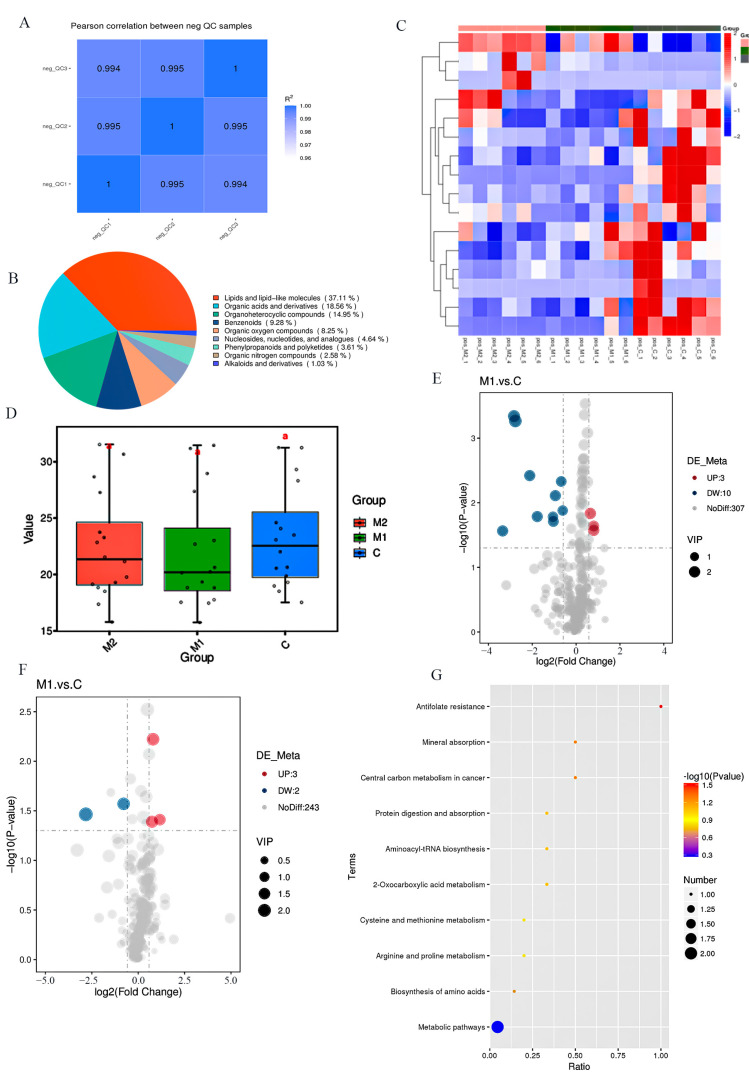
Milk metabolome analyses (the POS mode). (**A**) Calculation of Pearson correlation coefficients between QC samples based on relative quantitative values of metabolites. (**B**) Pie chart of metabolite level 1 classification. (**C**) Clustering heat map analysis of differential metabolites in the total cluster. (**D**) Box plot: the vertical coordinate is the metabolite mean value, and the horizontal coordinate is the grouping of the samples; “a” is the result of a significant difference analysis. (**E,F**) Volcanic map of differential metabolites: horizontal coordinates indicate a fold change in metabolite differences across subgroups, and vertical coordinates indicate a significant level of differences. (**G**) KEGG enrichment bubble diagram: the horizontal coordinates are x/y, the color of the point represents the *p*-value of the hypergeometric test, and the size of the point represents the number of differential metabolites in the corresponding pathway.

Quality control (QC) was performed on the data (Figure 1A). A total of 568 metabolites, 320 in the positive mode (POS) and 248 in the negative mode (NEG) were detected in donkey milk. The most prevalent metabolites in donkey milk were lipids and lipid-like molecules, organic acids and derivatives, and organoheterocyclic compounds (Figure 1B). The differential metabolites among groups C, M1, and M2 were analyzed. The clustering heat map of metabolites is shown in Figure 1C. Metabolites such as 2-[(3S)-1-Benzyl-3-pyrrolidinyl]-1-methyl-1H-benzimidazole in M1 v C and Bicyclo[2.2.2]oct-2-en-1-yl4-methylbenzene-1-sulfonate in M2 v C were significantly different. Figure 1D shows the metabolite numbers among the different groups. A total of 41 metabolites were found to be differential metabolites, 16 of which were up-regulated and 25 of which were down-regulated, as shown in Figure 1E,F. Twelve distinct metabolites were down-regulated, and six were up-regulated in M1 v C. Ten differential metabolites were up-regulated, and thirteen were down-regulated in M2 v C. The differential metabolites between different groups are shown in Table 4 and Table 5. Nine differential metabolites were found between the Met-supplemented groups and the control group (Table 4). The addition of Met significantly increased 2-(3,4-dihydroxyphenyl) acetamide, n-(1,3-benzodioxol-5-yl)-7-chloroquinolin-4-amine, 23-Nordeoxycholic acid, and LPA 10:0 (*p* < 0.05). Ten differential metabolites were found between groups M1 and group C, and 14 differential metabolites were found between groups M2 and group C (Table 5), and the differential metabolites matched the differential metabolite species of the clustered heat map in Figure 1C. In the M1 v C and M2 v C comparisons, the main differential metabolites were Met and D-(+)-proline. The relevant metabolic pathways involved were as follows: antifolate resistance, mineral absorption, central carbon metabolism in cancer, aminoacyl-tRNA biosynthesis, 2-oxocarboxylic acid metabolism, protein digestion and absorption, cysteine and Met metabolism, arginine and proline metabolism, and amino acid biosynthesis. Among them, there were significant differences in the metabolic pathway of antifolate resistance (*p* < 0.05). (Figure 1G).

### 3.3. Effects of Met Supplementation on Donkey Serum Metabolites

**Figure 2:**
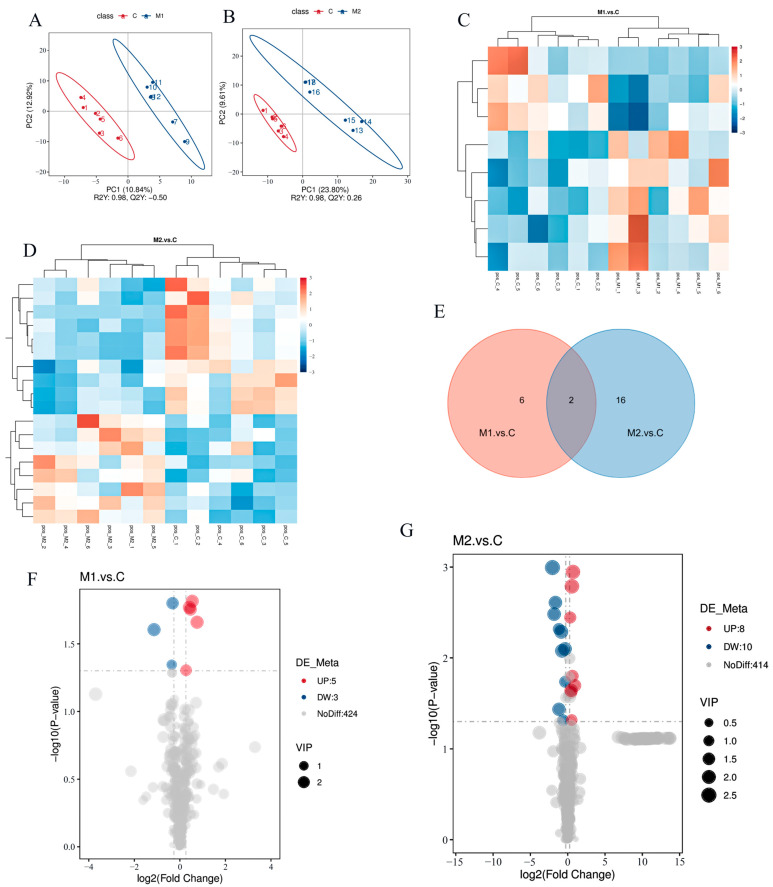
Serum metabolome analyses (the above figure only shows the results of the analysis in the POS mode). (**A**,**B**) PLSDA plot of M1 vs. C and M2 vs. C. (**C**,**D**) Hierarchical cluster analyses were performed on the obtained differential metabolites of the two groups to derive the differences in metabolic expression patterns between and within the two groups for the same pair of comparisons. (**E**) Venn diagram of differential metabolites of M1 vs. C and M2 vs. C. (**F**,**G**) Differential metabolite volcano plot of M1 vs. C and M2 vs. C.

Serum metabolism data were analyzed using the PLSDA method (Figure 2A,B). The clustering heat map was used for the hierarchical clustering analysis of differential metabolites (Figure 2C,D). The Venn plots show the number of differential metabolites between different groups (Figure 2E). As shown in Figure 2F,G, 753 metabolites were identified in the serum of lactating donkeys, 432 in the POS mode, and 321 in the NEG mode, with 65 distinct metabolites found. Six differential metabolites were down-regulated, and eleven were up-regulated in M1 v C (Figure 2F). Figure 2G shows how, from the divergent metabolites in M2 v C, 15 were up-regulated, and 33 were down-regulated. Table 6 and Table 7 present the differential metabolites.

**Figure 3:**
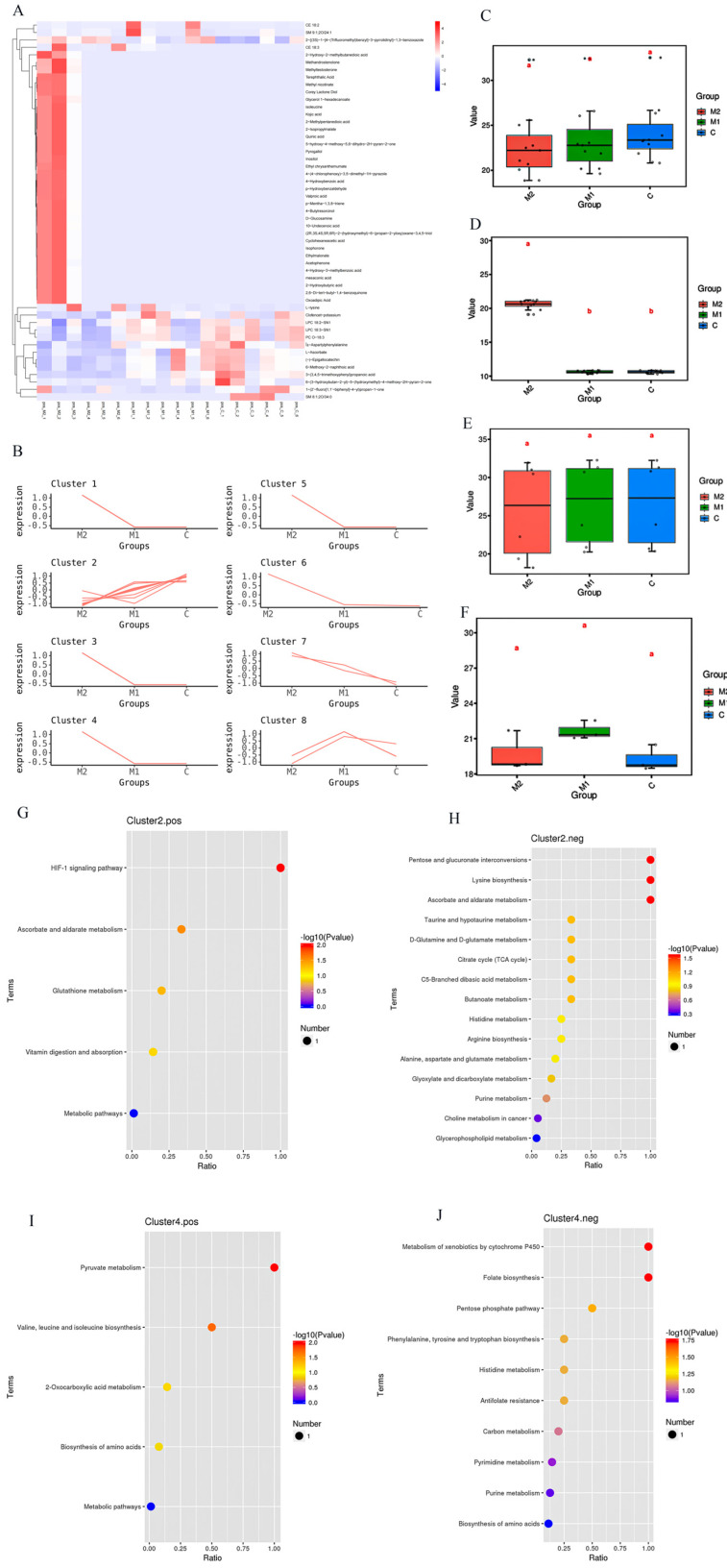
ANOVA analyses (some of the results of the analyses are presented in the figure above). (**A**) Cluster heat map. The vertical line represents samples, the color bar represents sample grouping information, and the horizontal line represents metabolites. (**B**) K-Means plot for clustering. The horizontal coordinate is the name of the sample group, and the vertical coordinate is the value after the z-score of the relative metabolite content. A cluster represents a cluster of metabolites with the same trend. (**C**–**F**) Box-and-line plots; “a” and “b” are the result of a significant difference analysis. The vertical coordinate is the mean metabolite value, and the horizontal coordinate is the grouping of the samples. (**G**–**J**) Differential metabolite enrichment bubble plots. The vertical coordinate is the mean metabolite value, and the horizontal coordinate is the grouping of the samples.

The clustering analysis of the differential metabolites is displayed in Figure 3A,B. Differential metabolites obtained from different cluster comparisons were box-and-line-plotted to show the changes and differences in differential metabolites in the M1, M2, and C groups (Figure 3C–F). The KEGG enrichment bubble plots were analyzed after clustering (Figure 3G–J). The levels of the differential metabolite Inositol were significantly higher in Inositol phosphate metabolism and the phosphatidylinositol signaling system. Biotin metabolism, lysine degradation, and lysine biosynthesis significantly increased in l-lysine, while l-ascorbate significantly increased in ascorbate, aldarate, and glutathione metabolism.

## 4. Discussion

### 4.1. Effects of Met Supplementation on Donkey Lactation Performance

In dairy cows, the addition of Met can increase the synthesis of milk proteins and increase milk output [23,24]. In a separate study of lactating female donkeys fed alfalfa instead of poor-quality roughage, an increase in lysine was found, which was reflected by the weight gain of the foals [25]. Research revealed that supplementing dairy cows with rumen-protected Met led to considerable increases in both milk output and the milk protein rate [26,27,28]. Comparable findings indicate that adding rumen-protected Met to the diet increases the protein content of milk [29]. According to research conducted on Awassi ewes, adding 5 g of rumen-protected Met to their diet increased the amount of casein and milk protein in the milk [30]. In the present study, the addition of 5 g of Met significantly increased the milk yield and milk component yield of lactating donkeys, and considerable (*p* < 0.05) weight gain was observed in the foals. Thus, our findings align with previous studies and findings on dairy cows.

### 4.2. Effect of Met Addition on Milk Metabolites

Nutrients alter metabolites in milk [31]. Furthermore, the addition of Met significantly increased LPS16:0 (M1 group) and L-aspartic acid (M2 group) in donkey milk. L-aspartic acid is a competitive inhibitor of β-glucuronidase [32]; infants consuming casein hydrolysate formulas had lower levels of jaundice because casein hydrolysates contain β-glucuronidase inhibitors, and the addition of L-aspartic acid could theoretically be effective in preventing a reduction in jaundice levels. Metabolomics testing of human milk revealed significant increases in the levels of LPS 16:0, myristic, and L-aspartic acids. While donkey milk is a good candidate for infant formula on its own [7], the addition of Met increased the levels of L-aspartic acid, which is an amino acid, and myristic acid, which is an important fatty acid, that improves the quality and tolerance of infant formulas [33]. A study on the addition of small molecules to dairy goat diets revealed that the addition of either 2 g or 4 g of isoquinoline alkaloid berberine increased milk production [34]. Changes in the production of mammary fat in milk were observed after the addition of puffed linseed and breed-combined puffed linseed to goat milk diets [35]. In the present study, the addition of Met altered the metabolites in milk. The major differential metabolites identified in this study impact antifolate resistance and the biosynthesis of amino acid pathways.

### 4.3. Effect of Met Addition on Serum Metabolites

Blood composition affects milk production and milk composition in lactating animals [36]. Donkey milk is more similar to human milk than that of cows. Donkey milk also has greater ascorbate levels, which increase when methionine is added to a donkey’s diet compared to cow milk [5]. The addition of 15 g of Met resulted in a significant up-regulation of L-ascorbate in the HIF-1 signaling pathway, whereas 5-phosphoribosyl 1-pyrophosphate, involved in antifolate resistance, was significantly down-regulated. Research indicates that supplementing dairy cow diets with rumen-protected capsicum oleoresin during lactation affects their blood metabolites (glucose, urea-N, non-esterified fatty acids, and β-hydroxybutyric acid), which results in an increase in milk production [37]. The addition of calcareous marine algae to the diet of Holstein dairy cows affected serum Na concentrations and increased milk protein production [38]. A diet supplemented with rumen-protected lysine (RPL) was found to improve lactation performance in dairy cows and can increase milk component production when added to postpartum cow diets [39]. In this pilot study, 5 g of Met was added to the diets of lactating donkeys, which led to a notable up-regulation of leukotriene C4 in the Fc epsilon RI signaling pathway and a significant down-regulation of vitamin B2 in riboflavin metabolism. Research has shown that the HIF-1 signaling pathway is associated with neutral lipid metabolism for intramuscular fat synthesis in the Guangling donkey [40]. The addition of 5 g of Met significantly increased L-lysine in biotin metabolism, lysine degradation, and lysine biosynthesis. L-ascorbate was also significantly up-regulated, as was inositol in phosphate metabolism. Met increased milk yield, milk protein, and milk fat production by increasing serum levels of L-ascorbate, inositol, and L-lysine.

## 5. Conclusions

The current study investigated the processes and effects of Met addition on donkey lactation performance. The results demonstrate that the addition of 5 g of Met to the diet caused a considerable increase in milk output, the production of milk proteins, lactoferrin and milk fat, and milk metabolites, such as myristic acid, d-glutamine, l-aspartic acid, and LPS 16:0. The addition of Met resulted in the up-regulation of blood metabolites, such as L-ascorbate, inositol, and L-lysine. In conclusion, Met increased donkey milk production and milk composition yields and improved milk metabolites by regulating serum metabolites. This is the first study on the regulatory effect of Met on donkey milk synthesis, which provides a foundation for the milk production performance improvement of lactating donkeys and the development and utilization of donkey milk.

## Figures and Tables

**Table 1:** The concentrate composition and nutrient levels of experimental diets.

Ingredients, %	C	M1	M2
Corn	40.60	40.60	40.60
Soybean meal	27.00	27.00	27.00
Wheat bran	21.00	21.00	21.00
Stone flour	6.30	6.30	6.30
Calcium hydrogen phosphate	1.60	1.60	1.60
Salt	0.70	0.70	0.70
Sodium bicarbonate	0.50	0.50	0.50
Vitamin complex	0.10	0.10	0.10
Mineral complex	1.00	1.00	1.00
Choline chloride	0.30	0.30	0.30
Lysine	0.90	0.90	0.90
Met (added)	0	0.002	0.006
Chemical composition, %			
Dry matter	87.80	87.80	87.80
Crude protein	18.20	18.20	18.20
Crude fiber	3.73	3.73	3.73
Crude ash	12.02	12.02	12.02
Crude fat	2.79	2.79	2.79
Calcium	0.74	0.74	0.74
Total phosphorus	0.74	0.74	0.74
Non-phytate phosphorus	0.43	0.43	0.43
Na	0.76	0.76	0.76
Lysine	1.38	1.38	1.38

Note: C, control group, Met 0 g/d; M1, Met group I, Met 5 g/d; and M2, Met group II, Met 15 g/d. Met (added), or Met in the ingredient list, is the weight of additional Met added as a percentage of the total concentrate feed.

**Table 2:** Effects of Met supplementation on donkey milk yield and foals’ weight gain.

Item	C	M1	M2	*p*-Value
Milk yield (kg)	1.15 ± 0.22 ^b^	1.72 ± 0.23 ^a^	1.14 ± 0.32 ^b^	0.0054
Weight gain of foals (kg)	15.1 ± 2.7 ^b^	19.4 ± 4.1 ^ab^	19.6 ± 2.7 ^a^	0.0305

Note: Means within the same row without the same superscripts differ significantly at *p* < 0.05. The same is true below.

**Table 3:** Effects of Met supplementation on milk composition content and yield of Dezhou donkey.

Item	C	M1	M2	*p*-Value
Milk composition content				
Protein (%)	1.51 ± 0.12 ^b^	1.55 ± 0.06 ^b^	1.71 ± 0.11 ^a^	0.0240
Fat (%)	0.40 ± 0.44	0.30 ± 0.09	0.24 ± 0.08	0.0802
Lactose (%)	6.63 ± 0.20	6.72 ± 0.11	6.63 ± 0.10	0.5299
Lactoferrin (g/L)	0.094 ± 0.0017 ^b^	0.097 ± 0.0008 ^a^	0.097 ± 0.0011 ^a^	0.0040
PFA (%)	0.1987 ± 0.0064 ^b^	0.2126 ± 0.0068 ^a^	0.2084 ± 0.0058 ^a^	0.0134
Solid (%)	9.04 ± 0.10 ^b^	9.27 ± 0.08 ^a^	9.32 ± 0.12 ^a^	0.0022
SNF (%)	8.90 ± 0.17 ^b^	9.04 ± 0.11 ^ab^	9.13 ± 0.06 ^a^	0.0313
Urea (mg/dL)	14.83 ± 0.80	14.05 ± 1.71	14.48 ± 0.80	0.5884
Milk composition yield				
Protein (g)	16.62 ± 2.75 ^b^	25.18 ± 3.26 ^a^	18.79 ± 6.02 ^ab^	0.0208
Fat (g)	1.88 ± 1.01 ^b^	4.82 ± 2.48 ^a^	2.69 ± 1.14 ^ab^	0.0445
Lactose (g)	73.10 ± 14.27 ^b^	110.26 ± 13.49 ^a^	71.87 ± 19.33 ^b^	0.0035
Lactoferrin (mg)	10.35 ± 1.86 ^b^	15.88 ± 2.07 ^a^	10.61 ± 3.00 ^b^	0.0046
PFA (mg)	2.19 ± 0.41 ^b^	3.43 ± 0.46 ^a^	2.27 ± 0.65 ^b^	0.0039
Solid (g)	99.69 ± 18.51 ^b^	152.40 ± 20.20 ^a^	101.77 ± 28.54 ^b^	0.0049
SNF (g)	97.99 ± 17.71 ^b^	148.43 ± 17.77 ^a^	99.64 ± 27.8 ^b^	0.0045

Note: Means within the same row without the same superscripts differ significantly at *p* < 0.05.

**Table 4:** Differences in metabolites between Met addition groups and the control group.

Differential Metabolites	FC	VIP	*p*-Value	ROC	Up. Down
N-(1,3-benzodioxol-5-yl)-7-chloroquinolin-4-amine	1.61	2.12	0.0089	0.97	up
23-Nordeoxycholic acid	1.54	1.55	0.0178	0.89	up
LPA 10:0	1.78	2.01	0.0257	0.86	up
2-(3,4-dihydroxyphenyl)acetamide	1.79	2.02	0.0014	0.97	up
4-oxododecanedioic acid	0.42	1.80	0.0063	0.94	down
Met	0.06	2.70	0.0000	1.00	down
SM9:1;2O/32:7	0.15	2.57	0.0005	1.00	down
D-(+)-proline	0.48	1.87	0.0007	0.97	down
Thromoboxane B1	0.12	2.63	0.0285	1.00	down

Note: FC > 1.5 or FC < 0.667. The area under the receiver operating characteristic (ROC) curve is referred to as the area under the curve (AUC), with some predictive accuracy when the AUC value is between 0.7 and 0.9 and higher predictive accuracy when the AUC value is above 0.9. The following table is shown above.

**Table 5:** The differential metabolites between different groups.

Groups	Differential Metabolites	FC	VIP	*p*-Value	ROC	Up. Down
M1 v C	2-(4-aminophenoxy)isophthalonitrile	1.74	2.05	0.0267	0.91	up
	LPS16:0	2.24	1.56	0.0388	0.83	up
	2-[(3S)-1-Benzyl-3-pyrrolidinyl]-1-methyl-1H-benzimidazole	0.23	1.97	0.0037	0.94	down
	N2-Methylguanosine	0.62	1.74	0.0046	0.97	down
	RPK	0.65	1.63	0.0131	0.88	down
	INK	0.29	1.96	0.0163	0.91	down
	ELK	0.48	1.82	0.0194	0.86	down
	trans,trans-2,4-Heptadienal	0.09	1.97	0.0272	0.88	down
	8-iso-15-ketoProstaglandin F2α	0.57	1.94	0.0269	0.83	down
	Acetyl-L-carnitine	0.63	2.25	0.0046	1.00	down
M2 v C	Bicyclo[2.2.2]oct-2-en-1-yl4-methylbenzene-1-sulfonate	12.39	2.30	0.0093	1.00	up
	2-Aminobenzenesulfonic acid	1.99	1.83	0.0157	0.88	up
	FLK	2.12	1.30	0.0434	0.83	up
	Benzoic acid	1.63	2.09	0.0106	0.91	up
	Myristic acid	2.04	1.84	0.0246	0.88	up
	L-aspartic Acid	1.68	1.99	0.0385	0.86	up
	LPC18:0-SN1	0.57	1.87	0.0135	0.86	down
	Kynurenic acid	0.48	1.83	0.0226	0.97	down
	α-Hydroxyhippuric acid	0.53	1.69	0.0494	0.77	down
	3-Hydroxybutyric acid	0.52	2.97	0.0022	0.91	down
	2-Hydroxyhippuric acid	0.46	2.26	0.0182	0.94	down
	3-Methylbutyroylcarnitine	0.47	1.87	0.0382	0.88	down
	4-Hydroxy-3-methoxyphenylglycol sulfate	0.27	2.47	0.0468	0.86	down

**Table 6:** Differences in metabolites when comparing M2 v M1 v C.

Differential Metabolites	FC	VIP	*p*-Value	ROC	Up. Down
1-(4-benzylpiperazino)-yridinedin-2-ylamino)propan-1-one	1.38	2.28	0.0175	0.91	up
4-(4-nitrophenylazo)aniline	1.68	2.64	0.0218	0.83	up
1-Stearoyl-2-linoleoyl-sn-glycero-3-phosphoethanolamine(PEO-18:0_18:2)	0.56	1.73	0.0119	0.97	down
5-Sulfosalicylic acid	0.54	2.55	0.0203	0.83	down
Vitamin B2	0.71	1.57	0.0246	0.88	down

**Table 7:** The differential metabolites between the M1 and C groups as well as the M2 and C groups.

Groups	Differential Metabolites	FC	VIP	*p*-Value	ROC	Up. Down
M1 v C	LPE O-16:1	1.70	2.54	0.0026	0.97	up
	LPE O-17:1	1.58	2.29	0.0071	0.94	up
	SM8:0;2O/20:0	5.96	2.77	0.0086	0.91	up
	LPC O-18:2	1.27	1.76	0.0134	0.86	up
	LPC O-17:1	1.56	2.24	0.0226	0.89	up
	Leukotriene C4	1.55	2.02	0.0252	0.86	up
	N-(1,3-benzodioxol-5-yl)-7-chloroquinolin-4-amine	1.46	2.57	0.0153	0.89	up
M2 v C	CAR 5:0	1.33	2.57	0.0170	0.92	up
	LPC O-18:1	1.21	1.90	0.0495	0.75	up
	SM 8:1;2O/30:0	0.80	2.23	0.0158	0.89	down
	1-(2′-fluoro[1,1′-biphenyl]-4-yl)propan-1-one	0.45	2.63	0.0248	0.89	down
	PC 17:0_18:1	0.78	1.34	0.0451	0.89	down
	2-(1H-benzimidazol-2-yl)-N-[4-(benzyloxy) phenyl] benzamide	1.47	2.29	0.0016	0.94	up
	methyl 2-(acetylamino)-4-amino-4-oxobutanoate	1.26	1.36	0.0035	0.94	up
	ST27:1;O;S	1.84	2.34	0.0000	1	up
	(-)-Epigallocatechin	0.31	1.89	0.0024	0.97	down
	6-Methoxy-2-naphthoic acid	0.28	1.93	0.0032	0.97	down
	3-(3,4,5-trimethoxyphenyl)propanoic acid	0.24	2.50	0.0013	1	down
	L-Ascorbate	0.43	1.35	0.0048	0.94	down
	PC O-18:3	0.55	2.01	0.0051	0.91	down
	Phosphocholine	0.78	1.89	0.0079	0.91	down
	LPC18:3-SN1	0.58	1.97	0.0083	0.91	down
	N2-(2-methoxyphenyl)-5-chloro-3-methylbenzo[b]thiophene-2-sulfonamide	0.22	2.41	0.0001	1	down
	LPC20:3	0.77	1.76	0.0015	1	down
	LPC18:3	0.53	2.59	0.0023	0.94	down
	Glycolithocholic acid	0.39	1.77	0.0071	0.94	down

## Data Availability

The data were not deposited in a public repository. The data are available upon reasonable request directly from the corresponding authors.

## References

[1] Renu G., Arun B., Karnam S., Rahul M., Yash P., Varij N., Mir Asif I., Sarika J., Harish K. (2023). Milk from Halari Donkey Breed: Nutritional Analysis, Vitamins, Minerals, and Amino Acids Profiling. Foods.

[2] Vincenzo L., Aristide M., Angela S., Salvatore C., Pasquale De P., Domenico R., Giuseppina P., Gianluca N. (2021). Evaluation of Different Test-Day Milk Recording Protocols by Wood’s Model Application for the Estimation of Dairy Goat Milk and Milk Constituent Yield. Animals.

[3] Fantuz F., Ferraro S., Todini L., Cimarelli L., Fatica A., Marcantoni F., Salimei E. (2020). Distribution of calcium, phosphorus, sulfur, magnesium, potassium, and sodium in major fractions of donkey milk. J. Dairy Science.

[4] Li M., Dong Y., Li W., Shen X., Abdlla R., Chen J., Cao X., Yue X. (2022). Characterization and comparison of whey proteomes from bovine and donkey colostrum and mature milk. Lebensm.-Wiss. Technol..

[5] Vincenzetti S., Santini G., Polzonetti V., Pucciarelli S., Klimanova Y., Polidori P. (2021). Vitamins in Human and Donkey Milk: Functional and Nutritional Role. Nutrients.

[6] Suzely A.S.M., Marcelo A.A., Cléa A.S.G., Tânia A.S., Orlando S. (2018). Caries in children with lactose intolerance and cow’s milk protein allergy. Braz. Oral Res..

[7] Souroullas K., Aspri M., Papademas P. (2018). Donkey milk as a supplement in infant formula: Benefits and technological challenges. Food Res. Int..

[8] David H., Ralf G.H., Donald J.S.C., Anthony G.C.S., Chung W.C., Dorothy F., Clifford S.H. (2000). Role of food protein intolerance in infants with persistent distress attributed to reflux esophagitis. J. Pediatr..

[9] Cresi F., Maggiora E., Pirra A., Tonetto P., Rubino C., Cavallarin L., Giribaldi M., Moro G.E., Peila C., Coscia A. (2020). Effects on Gastroesophageal Reflux of Donkey Milk-Derived Human Milk Fortifier Versus Standard Fortifier in Preterm Newborns: Additional Data from the FortiLat Study. Nutrients.

[10] Silvia V., Stefania P., Giuseppe S., Yulia K., Valeria P., Paolo P. (2020). B-Vitamins Determination in Donkey Milk. Beverages.

[11] Renu G., Karnam S., Rahul M., Anurag B., Yash P., Varij N., Legha R.A., Manish T., ManMohan Singh C., Mir Asif I. (2023). Comparative metabolomics analysis of Halari donkey colostrum and mature milk throughout lactation stages using 1H Nuclear Magnetic Resonance. Lebensm.-Wiss. Technol..

[12] Yue Y., Li L., Tong M., Li S., Zhao Y., Guo X., Guo Y., Shi B., Yan S. (2022). Effect of Varying Dietary Crude Protein Level on Milk Production, Nutrient Digestibility, and Serum Metabolites by Lactating Donkeys. Animals.

[13] Isabela Claudia Barbosa dos S., Adriano Henrique do Nascimento R., Cláudio Vaz Di Mambro R., Chiara Albano de Araújo O. (2023). Donkey milk composition is altered by lactation stage and jennies age. J. Food Compos. Anal..

[14] Ante I., Gordan Š., Giovanni B., Edmondo Š., Nicolò A., Jasna A., Nikolina Kelava U., Lana P., Mateja P., Miljenko K. (2023). Potential of Endangered Local Donkey Breeds in Meat and Milk Production. Animals.

[15] Mohsen Danesh M., Hassan K., Rieke J., Sadjad Danesh M., Aghil G., Amir Mansour V. (2022). Rumen-protected zinc–methionine dietary inclusion alters dairy cow performances, and oxidative and inflammatory status under long-term environmental heat stress. Front. Vet. Sci..

[16] Toledo M.Z., Baez G.M., Garcia-Guerra A., Lobos N.E., Guenther J.N., Trevisol E., Luchini D., Shaver R.D., Wiltbank M.C. (2017). Effect of feeding rumen-protected methionine on productive and reproductive performance of dairy cows. PLoS ONE.

[17] Emrah G., EkİN S. (2022). Effects of rumen-protected methionine and lysine on milk yield and milk composition inHolstein dairy cows consuming a corn grain and canola meal-based diet. Turk. J. Vet. Anim. Sci..

[18] Zanton G.I., Bowman G.R., Vázquez-Añón M., Rode L.M. (2014). Meta-analysis of lactation performance in dairy cows receiving supplemental dietary methionine sources or postruminal infusion of methionine. J. Dairy Sci..

[19] Liang S.L., Wei Z.H., Wu J.J., Dong X.L., Liu J.X., Wang D.M. (2019). Effect of N-acetyl-l-methionine supplementation on lactation performance and plasma variables in mid-lactating dairy cows. J. Dairy Sci..

[20] Wang J., Zhang C., Zhao Q., Li C., Jin S., Gu X. (2020). Metabolic Profiling of Plasma in Different Calving Body Condition Score Cows Using an Untargeted Liquid Chromatography-Mass Spectrometry Metabolomics Approach. Animals.

[21] Heischmann S., Quinn K., Cruickshank-Quinn C., Liang L.P., Reisdorph R., Reisdorph N., Patel M. (2016). Exploratory Metabolomics Profiling in the Kainic Acid Rat Model Reveals Depletion of 25-Hydroxyvitamin D3 during Epileptogenesis. Sci. Rep..

[22] Rao G., Sui J., Zhang J. (2016). Metabolomics reveals significant variations in metabolites and correlations regarding the maturation of walnuts (*Juglans regia* L.). Biol. Open.

[23] Zhang J., Deng L., Zhang X., Cao Y., Li M., Yao J. (2023). Multiple essential amino acids regulate mammary metabolism and milk protein synthesis in lactating dairy cows. Anim. Feed. Sci. Technol..

[24] Wen X., Ning H., Liu H.Y. (2023). Supplementation of Methionine Dipeptide Enhances the Milking Performance of Lactating Dairy Cows. Animals.

[25] Liang X.S., Yue Y.X., Zhao Y.L., Guo Y.M., Guo X.Y., Shi B.L., Yan S.M. (2022). Effects of dietary concentrate to forage ratio on milk performance, milk amino acid composition and milk protein synthesis of lactating donkeys. Anim. Feed. Sci. Technol..

[26] Wei C., He T., Wan X., Liu S., Dong Y., Qu Y. (2022). Meta-Analysis of Rumen-Protected Methionine in Milk Production and Composition of Dairy Cows. Animals.

[27] Swanepoel N., Paul H.R., Alan J.C. (2020). Impacts of substitution of canola meal with soybean meal, with and without ruminally protected methionine, on production, reproduction and health of early lactation multiparous Holstein cows through 160 days in milk. Anim. Feed. Sci. Technol..

[28] Valdir C., Fernanda L., Schwab C.G., Mateus Z.T., Edgar Alain C.-S. (2020). Effects of rumen-protected methionine supplementation on the performance of high production dairy cows in the tropics. PLoS ONE.

[29] Zhang Y., Sina Saed S., Tager L.R., McFadden J.W., Krause K.M. (2016). Comparative effects of multiple sources of rumen-protected methionine on milk production and serum amino acid levels in midlactation dairy cows. J. Anim. Sci..

[30] Titi H.H., Mufeed A.A., Mohamed A.A.-M. (2022). Effect of supplemental rumen-protected methionine on reproduction and production of Awassi ewes. Ital. J. Anim. Sci..

[31] Gu F., Liang S., Zhu S., Liu J., Sun H.Z. (2021). Multi-omics revealed the effects of rumen-protected methionine on the nutrient profile of milk in dairy cows. Food Res. Int..

[32] Bill L.K., Frank L.S., Glenn R.G. (2001). A Novel Inhibitor of β-Glucuronidase: L-Aspartic Acid. Pediatr. Res..

[33] Livia Simon S., Miaomiao Z., Géza M., Réka A.V., Oksana M., Eszter B., Sandor G.V. (2022). Fatty Acid Composition of Milk from Mothers with Normal Weight, Obesity, or Gestational Diabetes. Life.

[34] Navid G., Nasri M.H.F., Seyyed Homayoun F., Seyyed Ehsan G., Einar V.B.P. (2021). Regulation of Nutritional Metabolism in Transition Dairy Goats: Energy Balance, Liver Activity, and Insulin Resistance in Response to Berberine Supplementation. Animals.

[35] Bernard L., Christine L., Rouel J., Carole D., Kevin J.S., Yves C. (2015). Effect of extruded linseeds alone or in combination with fish oil on intake, milk production, plasma metabolite concentrations and milk fatty acid composition in lactating goats. Animal.

[36] Abeer M.E.-E., Ibrahim M.K., Abdou A., Abdel-Wahed A.M. (2019). Effect of Linseed oil Beads Addition with Vitamin E on Performance, Blood Metabolites and milk yield of lactating goats. Egypt. J. Nutr. Feed..

[37] Takiya C.S., Grigoletto N.T., Chesini R.G., Sbaralho O.P., Bugoni M., Vittorazzi P.C., Nunes A.T., da Silva G.G., Vieira D.J.C., de Freitas A.C. (2023). Feeding rumen-protected Capsicum oleoresin to dairy cows during the transition period and early lactation: Effects on nutrient digestibility, blood metabolites, and performance. Anim. Feed. Sci. Technol..

[38] Wu Z., Bernard J.K., Taylor S.J. (2015). Effect of feeding calcareous marine algae to Holstein cows prepartum or postpartum on serum metabolites and performance. J. Dairy Sci..

[39] Fehlberg L.K., Guadagnin A.R., Thomas B., Yasuro S., Shinzato I., Cardoso F.C. (2020). Feeding rumen-protected lysine prepartum increases energy-corrected milk and milk component yields in Holstein cows during early lactation. J. Dairy Sci..

[40] Li W., Qiu L., Guan J., Sun Y., Zhao J., Du M. (2022). Comparative transcriptome analysis of longissimus dorsi tissues with different intramuscular fat contents from Guangling donkeys. BMC Genom..

